# The Value of Plasma Paraquat Concentration in Predicting Therapeutic Effects of Haemoperfusion in Patients with Acute Paraquat Poisoning

**DOI:** 10.1371/journal.pone.0040911

**Published:** 2012-07-20

**Authors:** Yunying Shi, Yangjuan Bai, Yuangao Zou, Bei Cai, Fei Liu, Ping Fu, Lanlan Wang

**Affiliations:** 1 Department of Nephrology, West China Hospital of Sichuan University, Chengdu, People’s Republic of China; 2 Department of Laboratory Medicine, West China Hospital of Sichuan University, Chengdu, People's Republic of China; Indian Institute of Toxicology Reserach, India

## Abstract

**Objective:**

This study was aimed to analyze the scavenging effect of haemoperfusion on plasma paraquat (PQ) and to evaluate the clinical significance of PQ examination in the treatment of patients with acute paraquat poisoning.

**Methods:**

85 patients with acute paraquat intoxication by oral ingestion were admitted in West China Hospital from Jun, 2010 to Mar, 2011. A standardized therapeutic regimen including emergency haemoperfusion was given on all subjects. A total of 91 whole blood samples were taken before (0h), underway (1h after haemoperfusion beginning) and at the end (2h) of the haemoperfusion therapy. The clearance rate was calculated and related factors were analyzed.

**Results:**

As heamoperfusion was going on, the plasma paraquat concentration of the patients kept falling down. After 1 hour of haemoperfusion, the average clearance rate (R_1_) was 37.06±21.81%. After 2 hours of haemoperfusion, the average clearance rate (R_2_) was 45.99±23.13%. The average of R_1_/R_2_ ratio was 76.61±22.80%. In the high paraquat concentration group (plasma paraquat concentration (C_0_) >300 ng/mL), both the averages of R_1_ and R_2_ were significantly higher than those of the low paraquat concentration group (C_0_≤200 ng/mL) (p<0.05), and there was no significant difference of R_1_/R_2_ between these two groups (p>0.05).

**Conclusions:**

The dynamic monitoring of plasma PQ concentration was not only critical in the clinical evaluation but also helpful in guiding the treatment of patients with acute PQ intoxication. Haemoperfusion can effectively eliminate paraquat from the plasma in patients with high initial plasma PQ concentration, while in patients with low initial plasma PQ concentration (<200 ng/ml), the clearance effect of harmoperfusion was very limited. Increasing HP time might improve the overall clearance rate of HP on plasma PQ yet decrease the elimination efficiency of HP, while repeated HP treatment was helpful against the rebound phenomena.

## Introduction

Paraquat (PQ, 1,1′-dimethyl-4,4′-bipyridinium chloride) was a non-selective herbicide that has been widely used in countryside since the 1960s. Although it has been proved safe in occupational use, PQ poisoning has been observed in patients who ingest the pesticide either accidentally or intentionally as a suicide attempt. The mortality rate of PQ intoxication ranged from 50–90% and there was no specific antidote [1]. The most characteristic feature of PQ poisoning was lung damage, the mechanism of which lay in the selective accumulation of PQ in alveolar cells, inducing the production of large amount of toxic free radicals such as reactive oxygen species (ROS) which lead to lipid peroxidation of cell membrane, exhaustion of nicotinamide adenine dinucleotide phosphate (NADPH) and hence to cell death [2], [3], [4].

The clinical manifestations and outcomes of acute PQ intoxication are dependent on the exposure degree of PQ. Ingestion of PQ >20 ml of a 20% preparation is likely to cause death from multi-organ failure and cardiogenic shock within 1–4 days, while smaller quantities (10–20 ml) may initiate an irreversible lung fibrosis and renal failure that would result in death within several weeks. Therefore, the ingestion volume and plasma concentration of PQ were often used as important indicators of patients’ prognosis [5], [6], [7], [8], [9].

Extracorporeal elimination such as hemodialysis (HD) and haemoperfusion (HP) were common measurements used clinically in treating acute PQ intoxication. Many researchers believed that HP was more efficient in the clearance of plasma paraquat [10], [11], [12], [13]. Kang et al. proved that the PQ elimination by HP was as effective as or more effective than the renal elimination and inferred that early HP must be provided for life saving treatment in patients with acute PQ intoxication [14].

?twb .2w?>Recently, many studies focused on the relationship between HP and the overall prognosis of PQ poisoning. Unfortunately, the results were discouraging and controversial opinions aroused on the therapeutic effects of HP in the PQ intoxication treatment [15], [16]. However, due to the lack in standardized treatment regimen, it’s hard to make an evaluation objectively and factually. Meanwhile, limited studies was found focusing on the direct evaluation of the scavenging effect of HP on plasma PQ, most of which were in vitro simulation studies without dynamic in vivo data. Hence, doctors can only rely on their own clinical experiences and patients’ symptoms to perform HP, which was not beneficial for the development of such therapy or the improvement of the patients’ prognosis.

We have established an accurate and fast method for the quantitative detection of human plasma PQ concentration [17]. Therefore, in order to reveal the scavenging effects of HP on plasma PQ more directly and accurately, we plan to study the dynamic change of plasma PQ concentration during the HP process in patients with acute PQ poisoning, which might contribute to the theoretical basis of clinical regimen of acute PQ intoxication and the exploration of more efficient treatment. Meanwhile, we also hope to reveal the predictive significance of plasma PQ concentration monitoring in the treatment of patients with acute PQ intoxication.

## Materials and Methods

### Objectives

This study was aimed to analyze the scavenging effect of haemoperfusion on plasma paraquat (PQ) and to evaluate the clinical significance of PQ examination in the treatment of patients with acute paraquat poisoning.

### Participants

85 patients with acute paraquat intoxication by intended oral ingestion, who admitted to the Emergency Department of West China Hospital from Jun, 2010 to Mar, 2011, were included in this study (including 33 male and 52 female patients, with average age of 31.24±13.21 years). A detailed history taking, including demographic characteristics, past medical history, and specific questions about the paraquat poisoning (volume ingested, time of gastric lavage in local hospital and clinical symptoms) was completed. The exposure to PQ poisoning was assessed by the ingestion volume and a blood paraquat test. After checking vital signs, routine lab tests including completed blood cell counts, liver and renal function, coagulation function, arterial blood gas and routine urine analysis were conducted when admission. A unified therapeutic regimen including gastric lavage, fluid replacement, antioxidants (Vitamin C, Vitamin B and L-Glutathione) and immunosuppressant (corticosteroids) was given to all the patients. Then emergency haemoperfusion was performed on all the patients except ones who were diagnosed with multiple organ failure with unstable vital sign on admission. HP was conducted through a double lumen femoral venous catheter (Gambro, Germany) for 2 hours at a blood flow rate of 200 ml/min, using AK-200 haemoperfusion machine(Gambro, Germany)and a resin-containing column coated with polycarbonate (HA-230, Zhu Hai Jian Fan, China). We followed up these patients after they discharged from the hospital.

### Sample Collection

A set of three whole blood samples were withdrawn from poisoned patients and collected in heparinized tubes at the beginning (0h), underway (1h after haemoperfusion beginning) and the end (2h) of HP therapy, respectively, which were used for the examination of plasma PQ concentration and marked accordingly as C_0_, C_1_ and C_2_. 84 patients received emergent haemoperfusion, among whom 6 patients received repeated haemoperfusions (5 patients received twice and one patients received three times), thus there were totally 91 sets of samples.

### Examination of Plasma PQ Concentration

We used an improved approach for extraction and analysis of paraquat in human plasma [17]. A high performance liquid chromatographic (HPLC) system (Shimadzu, Japan) was used, using diethyl paraquat as an internal standard. The sample separation was achieved on a XtimateTM C18 column (250×4.6 mm, 5 um particle size)(Shimadzu, Japan). PQ and the internal standard (IS) were eluted under 35°C at a flow-rate of 1.0 ml/min and monitored by UV absorption at 256 nm. The mobile phase containing 150 mg sodium dodecyl sulfate (SDS) and 0.1 M orthophosphoric acid in 850 ml of deionized water was made, of which the pH was adjusted to 3.0 with the addition of triethylamine, and then acetonitrile was added to yield a 15% (v/v) proportion. Stock standard solutions (200 ug/ml) of PQ and IS were prepared in deionized water and stored at 4°C till use. The plasma samples (0.5 ml/sample) were spiked with 10 ul of known amounts of IS to yield a final concentration of 10 ug/ml. Samples were vortex-mixed after adding 1 ml acetonitrile and then centrifuged for 5 min at 12000 rpm in an Eppendorf 5810R Centrifuge (Eppendorfchina Ltd., Shanghai, China), Supernatants were transferred into a round bottom glass tube containing 3 ml methylene chloride, vortex-mixed for 5 min and centrifuged for 5 min at 2500 rpm. Finally, 100 ul upper aqueous phase was injected to HPLC analysis.

The detection limit (LOD) of this method was 10 ng/ml and the calibration curve obtained from extraction of plasma containing known amounts of PQ was linear over the quantities ranged from 20 ng/ml to 80000 ng/ml (R = 0.9999). The intra-day precision was less than 4.26%. The inter-day precision at all concentrations examined was less than 4.17%. The recovery rate of this method ranged from 88% to 115%.

### The Clearance Rate of Haemoperfusion on Plasma PQ (R)

We calculated the clearance rate according to the following formula: R_x_  = 100×(C_0_−C_x_)/C_0_ (%). R_x_ could be R_1_ or R_2_, representing the clearance rate of 1 hour HP or 2 hour HP on plasma PQ. C_0_ stood for the plasma PQ concentration at the beginning of HP treatment, while C_x_ stood for C_1_ or C_2_, representing the plasma PQ concentration at corresponding time point.

### Ethics Statement

The ethics committee of West China Hospital of Sichuan University specifically approved the study of “The value of plasma paraquat concentration in predicting therapeutic effects of haemoperfusion in patients with acute plasma paraquat poisoning”. All the 85 patients collected from Jun, 2010 to Mar, 2011 in this study were fully aware of the purpose and content of the study, and the clinical investigation was conducted according to the principles expressed in the Declaration of Helsinki. All the invasive therapeutic procedures were conducted with their voluntary permission and written informed consent. Because patients were all admitted from the emergency department in an urgent situation, and the test of plasma paraquat concentration was necessary to the treatment and prognosis, as well as no extra costs or procedures would be generated besides necessary treatment in the study, so the permission to the use of patients’ paraquat concentration data in this study were obtained by verbal informed consent instead of written form from all the patients, and all the data were analyzed anonymously. The ethics committee understood the situation and specifically approved such procedure.

### Statistical Analysis

The results were presented as mean ± standard error (X±SD). The differences among 3 groups were tested by one-way ANOVA while the comparison between 2 groups was achieved by Dunnett T3 or Tamhane’s T2. The data was analyzed using the Statistical Package for the Social Sciences version 14.0 (SPSS Inc, Chicago, IL, USA) statistical software. A p-value <0.05 was considered to be statistically significant.

## Results

Till May 2nd, 2011, 44 out of 85 patients died. The overall fatality rate was 48.24% and their medial survival time was 81.00 (2.00–201.50) days. The medial amount of PQ ingestion was 20 (10–50) ml. The average time from poisoning to gastric lavage was 0.5 (0.5–2.0) hour, while to the starting of HP was 6.75 (5.00–9.88) hours.

### The Change of Plasma PQ Concentration as Haemoperfusion Going on

**Figure 1:**
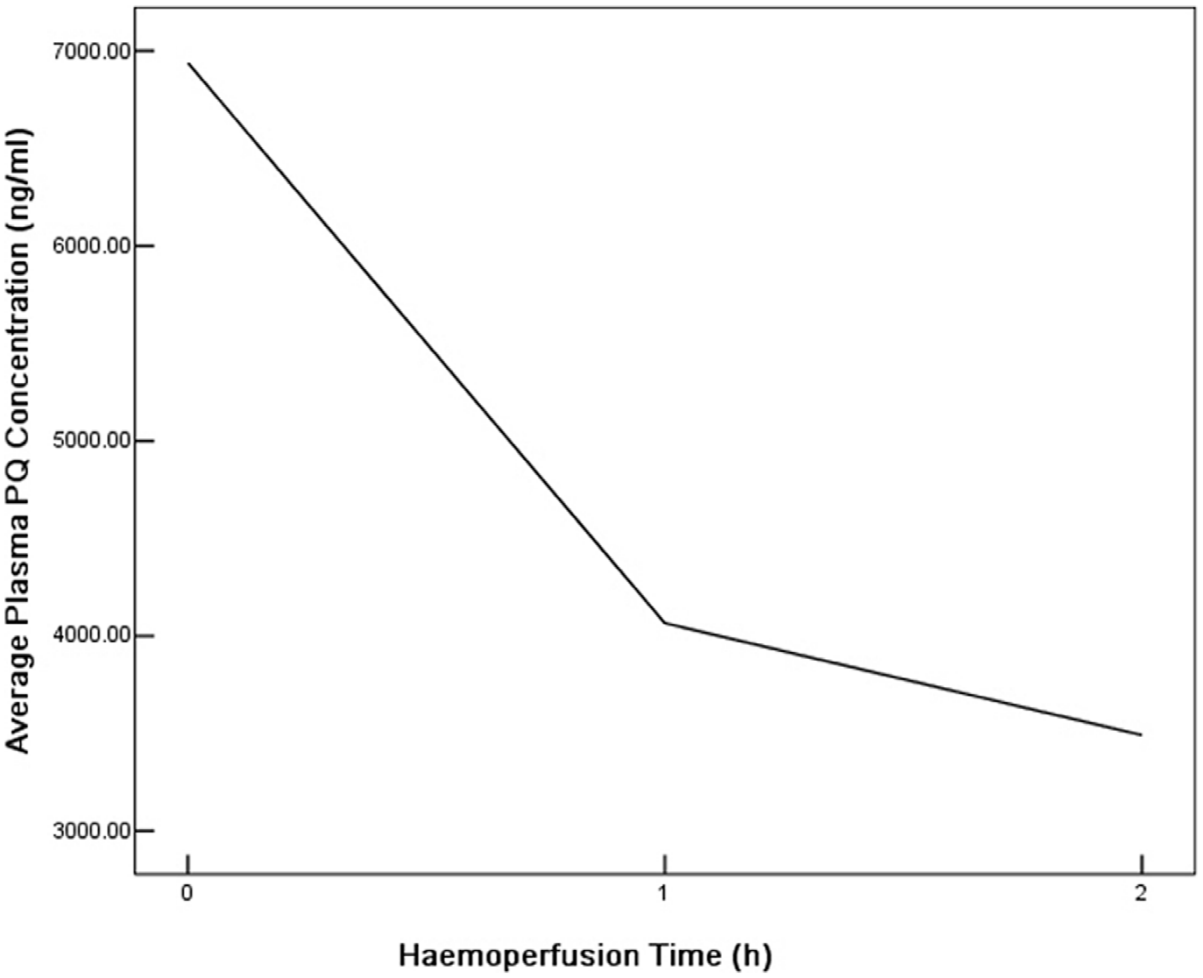
The change of average plasma PQ concentration as HP going on. The X axis stands for the treatment time of haemoperfusion (hour), while the Y axis stands for average plasma PQ concentration (ng/ml).

In our study, the medial plasma PQ concentration of the poisoned patients before HP treatment (C_0_) was 921.06 (201.57–5446.51) ng/ml. The average PQ concentration of all the 85 patients declined about 37.06±21.81% after 1 hour of HP treatment. Then the decrease of PQ concentration slowed down dramatically in the second hour. As shown by Figure 1, there was an obvious inflection point after the first hour. The average PQ clearance rate after 2 hours of HP (R_2_) was 45.99±23.13% and the average R_1_/R_2_ was 76.61±22.80%.

### The Relationship Between Plasma PQ Clearance Rate and C_0_

**Figure 2:**
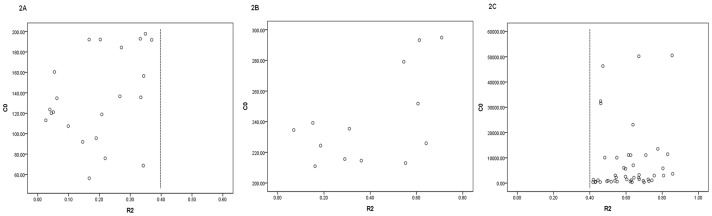
The relationship between C_0_ and the overall PQ clearance rate (R_2_) (C_0_<4000 **ng/ml).** C_0_ stands for the plasma PQ concentration at the beginning of haemoperfusion, R_2_ stands for the overall PQ clearace rate of haemoperfusion. Figure 2A stands for patients in group A with C_0_≤200 ng/ml; Figure 2B stands for patients in group B with 200<C_0_≤300 ng/ml; Figure 2C stands for patients in group C with C_0_>300 ng/ml.

By analyzing the plasma PQ clearance rate of patients with different C_0_ (Figure 2), we found that the PQ clearance rate (R_2_) of patients with C_0_ lower than 200 ng/mL were all below 40%, while for patients with C_0_ higher than 300 ng/mL, their R_2_ were all above 40%. Thus, according to C_0_, we classified the patients into 3 groups: group A with C_0_≤200 ng/mL, group C with C_0_>300 ng/mL and group B in between.

### The Comparison Among Three Groups with Different C_0_

No significant difference was found in gender, age, time from intoxication to gastric lavage, the time from intoxication to the starting of HP among three groups. As for ingestion volume, C1 and survival time, there were significant differences among 3 groups (Table 1). Patients in group A ingested much less PQ compared to patients in group B and C (p<0.05). C1 of patients in three groups followed a tendency as below: group C>group B>group A (p<0.05). The survival time of patients in group C were much shorter than that in group A and B (p<0.05).

**Table 1:** Basic Characteristics of patients with PQ poisoning.

	Group A	Group B	Group C
	(C0≤200)	(200<C0≤300)	(C0>300)
	N = 21	N = 10	N = 54
C1 (ng/ml) * ^ 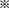 #^	99.80 (61.37–120.71)	196.90 (129.77–217.55)	1149.00 (546.49–5510.20)
Gender (Male%)	33.3	60.0	37.0
Age (yr)	29.19±13.01	32.90±15.65	31.72±12.98
Time to gastric lavage (hr)	1.00 (0.50–1.75)	1.25 (0.50–3.75)	0.50 (0.50–2.00)
Time to HP (hr)	9.00 (5.25–18.00)	7.00 (5.88–10.50)	6.00 (5.00–8.88)
Volume (ml) * #	10.00 (5.00–17.50)	30.00 (10.00–50.00)	30.00 (10.00–80.00)
Survival time (days) * 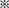	196.00 (153.00–271.00)	236.50 (173.50–273.50)	3.00 (1.75–78.75)

* : Comparison between Group A & C, p<0.05;

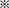
: Comparison between Group B & C, p<0.05;

# : Comparison between Group A & B, p<0.05.

Table 2 showed the average plasma PQ clearance rate in each group. Both R1 and R2 were the highest in group C and the lowest in group A, while the levels in group B were in between. R1 in group C was obviously higher than that in group A and B (p<0.05). Though R1 in group B was higher than that in group A, there was no significant difference (p>0.05). There was a statistical significance in R2 among 3 groups (p<0.05). No significant difference was found in R_1_/R_2_ among 3 groups (p>0.05). The relevance between R1, R2 and C0 was poor (r: 0.172∼0.332), so was the relevance between C_0_ and the clearance rate in each group (r: 0.076∼0.381).

**Table 2:** Comparison of PQ clearance rate among 3 groups (%).

Groups	N	R1* 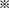	R2* ^ 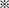 #^	R1/R2
Group A	26	16.38±11.18	20.39±12.41	75.01±25.82
(C_0_≤200)		(13.59, 5.67∼25.44)	(19.60, 5.61∼31.72)	(89.77, 56.42∼95.96)
Group B	13	27.66±18.14	39.97±22.01	63.90±30.75
(200<C_0_≤300)		(28.94, 9.53∼47.82)	(45.31, 21.15∼61.10)	(69.08, 36.29∼94.25)
Group C	53	49.30±16.41	60.19±14.48	81.39±14.42
(C_0_>300)		(51.62, 37.71∼59.84)	(61.35, 52.08∼67.04)	(84.85, 71.60∼92.66)

* : Comparison between Group A & C, p<0.05;

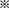
: Comparison between Group B & C, p<0.05;

# : Comparison between Group A & B, p<0.05.

### The Plasma PQ Concentrations of Patients Receiving Repeated HP

There were 6 patients in our study who received repeated HP. 5 of them received the second session of HP the next day, and one patient received HP once a day for continuous three days. The plasma PQ concentrations prior to the next session of HP (C_0–2_) of most patients (71.42%) were higher than their concentrations after the first session of HP (C_2–1_), while the rebound rates varied from −7.56% to 69.80% (Table 3).

**Table 3:** The plasma PQ concentrations of patients receiving repeated HP.

	C_0–1_	C_2–1_	C_0–2_	Rebound rate(%)
Patient 1	135.61	99.52	92.00	−7.56
Patient 2	235.42	162.48	231.23	42.31
Patient 3	279.11	127.00	215.64	69.80
Patient 4	399.63	278.00	192.13	−30.89
Patient 5	910.18	420.02	559.15	33.12
Patient 6-1	160.41	116.98	136.65	16.81
Patient 6-2	136.35	89.72	113.10	26.06

Note: C_0–1_ was the plasma PQ concentration prior to the first session of HP; C_2–1_ was the plasma PQ concentration after the first session of HP; C_0–2_ was the plasma PQ concentration prior to the second session of HP. PQ concentration : ng/mL; Rebound rate  =  (C_0–2_−C_2–1_)/C_2–1_.

## Discussion

Since the outcome of the patients with acute PQ intoxication is closely related to the plasma PQ concentration, the therapeutic effect of HP is closely associated with the elimination efficiency of HP on plasma PQ. The elimination efficiency of HP on plasma PQ was affected by many factors, while the haemoperfusion time was an important one among them. An in vitro study had reported that during the 2-hour HP treatment, the plasma PQ decreased dramatically as time went by (0.5, 1.0, 1.5h), but after 2 hours (2.5h, 3.0h), there was no obvious change in plasma PQ concentration anymore [7]. Considering the adverse effect and the limited advantage of long time HP, the HP time of the patients with acute PQ poisoning was set for 2 hours in our dialysis center. During the 2-hour process of haemoperfusion, the plasma PQ concentration of all the poisoned patients in our study declined continuously as HP going on,which inferred that PQ could be eliminated persistently from patients’ plasma during HP. After 2 hours of HP, the average clearance rate of plasma PQ (R_2_) was 45.99±23.13%. But the result of an in vitro study showed that the clearance rate of PQ after 2 hours of HP could reach 97.69∼99.52%, which was obviously higher than our data [18]. It inferred that the elimination ability of HP on plasma PQ differed from in vivo to in vitro. Thus, the direct observation on the elimination effect of HP on plasma PQ of poisoned patients is more significant and necessary in clinical guidance. Sae-Yong Honga et al. reported that the PQ clearance rate of patients in survival group after 4-hour HP was 80.3±19.9% compared to 67.2±19.2% in non-survival group, which was slightly higher than our result [10]. The prolonging of the perfusion time could increase the effective contact time between plasma toxin and resin particles, so that more toxins could be adsorbed on resin and then eliminated from plasma. Therefore, we inferred that different haemoperfusion time might be the reason to the difference in the overall PQ clearance rate between two studies. The advantage of long time HP was limited, because the complications such as imbalance of coagulative function and low blood pressure would increase and the elimination efficiency would decrease as the time went on. A recent in vitro study had reported that the elimination of HP on plasma could decrease from 215 ml/min at 30 min of HP to 22 ml/min at the 6^th^ hour [18]. Similar result was also found in our study. In our study, we also found that the clearance rate in the first hour of HP took up about three quarters of the overall PQ clearance rate and the decrease of PQ concentration slowed down dramatically in the second hour (Figure 1), which might infer that the elimination efficiency of HP was higher in the early phase and would decrease as time went on. At the beginning of HP, the resin particles were blank and their binding capacity was strong. The toxin can be adsorbed quickly and firmly by the resin particles so the plasma concentration decreased sharply. As the HP went on, the resin particles were gradually saturated and the decreasing speed of plasma PQ concentration slowed down. As we discussed latter in this paper, the elimination efficiency of HP might also be influenced by the plasma PQ concentration. In our study we found low elimination efficiency was associated with low plasma PQ concentration. Although the exact mechanism was unknown, the decrease of plasma PQ concentration after the early HP period might be one of the reasons for the decrease of the elimination efficiency in the later period. Therefore, although increasing HP time alone might improve the overall clearance rate of HP on plasma PQ, the elimination efficiency of HP would be decreased as time went on. Thus, further clinical studies are needed to find out the optimized therapeutic time of HP for patients with PQ poisoning so as to improve their prognosis and plasma PQ concentration monitoring will be of much help.

The plasma PQ concentration before HP treatment was another factor that might influence the elimination effect of HP on plasma PQ. The clearance rates of HP on plasma PQ at 1 hour and 2 hour in the group with highest pre-therapy concentration (group C) were both significantly higher than those in groups with lower pre-therapy concentrations (group A and group B, p<0.05). After 2 hours of HP treatment, the clearance rates of patients with pre-therapy PQ concentration below 200 ng/mL were all less than 40%, which were averagely about 20%. While for patients with pre-therapy PQ concentration above 300 ng/mL, whose average plasma PQ clearance rates were about 60% after 2 hours of HP (Figure 2). However, there was no linear correlation between the plasma clearance rate of HP and the plasma PQ concentration before HP treatment, especially when the initial PQ concentration was above 300 ng/mL. Therefore, the elimination ability of HP on plasma PQ was affected by the plasma PQ concentration before treatment. For patients with initial plasma PQ concentration above 300 ng/mL, HP could eliminate their plasma PQ efficiently, but for those with low initial concentration, the elimination effect of HP was quite limited. The average pre-HP plasma PQ concentration (C_0_) of patient group A was much lower than that of patient group B (p<0.05), but the survival time of patient group A wasn’t significantly longer than that of patient group B(p>0.05). These two groups of patients were comparable in age, gender, time from intoxication to gastric lavage, the time from intoxication to the starting of HP and were under the same therapeutic regimen, but the average plasma PQ clearance rate was significant lower in patient group A. Although the cause to such phenomenon was unclear, our result supplied that the ineffective elimination of HP on PQ might be a reason to the unsatisfactory therapeutic effect of patients with low PQ concentration. Thus, for those patients, alternative therapeutic measurements with better effectiveness should be considered prior to HP so as to improve the clinical outcome. It could be inferred from our study that in addition to the prognosis evaluation, the plasma PQ concentration monitoring is also helpful for the observation of therapeutic effect and the treatment selection.

In our study, 6 patients accepted HP for more than one time. In 5 out of the 6 patients, we found that the plasma PQ concentrations prior to the next HP were higher than their concentrations at the end of last HP respectively. Such rebound phenomena was also reported in an animal study [19], which inferred that PQ in patients’ tissues would reenter the blood circulation. Therefore, repeated HP treatment was required to remove toxin in the circulation of these patients. Although the rebound phenomena were found in most of the patients receiving repeated HP in our study (71.42%), this phenomena were not observed in two patients and the rebound rates also varied markedly among different patients (16.81% to 69.80%). That was to say not all the patients needed repeated HP. So the plasma PQ concentration must be monitored so as to help the physicians to evaluate the rebound phenomena and to determine whether repeated HP is needed.

In conclusion, the plasma PQ concentration monitoring is critical for the clinical treatment of PQ intoxication. The examination of plasma PQ concentration was not only critical in the clinical evaluation but also helpful in guiding the treatment of such patients. As for patients with initial plasma PQ concentration below 200 ng/ml, the clearance effect of HP was very limited and alternative therapeutic measurements with better effectiveness should be considered in priority so as to improve the clinical outcome. On the other hand, the dynamic monitoring of plasma PQ concentration can help physicians to identify rebound phenomena and decide whether repeated HP treatments are necessary. So the examination of plasma PQ concentration should be carried out as a routine clinical laboratory test. Further clinical studies were needed to find out the optimized therapeutic time and frequency of HP for patients with PQ poisoning so as to improve their overall prognosis under the help of plasma PQ concentration monitoring.
